# Identification of membrane-associated proteins with pathogenic potential expressed by *Corynebacterium pseudotuberculosis* grown in animal serum

**DOI:** 10.1186/s13104-018-3180-5

**Published:** 2018-01-25

**Authors:** José Tadeu Raynal, Bruno Lopes Bastos, Priscilla Carolinne Bagano Vilas-Boas, Thiago de Jesus Sousa, Marcos Costa-Silva, Maria da Conceição Aquino de Sá, Ricardo Wagner Portela, Lília Ferreira Moura-Costa, Vasco Azevedo, Roberto Meyer

**Affiliations:** 1 Laboratório de Imunologia e Biologia Molecular (LABIMUNO), Departamento de Biointeração, Instituto de Ciências da Saúde (ICS), Universidade Federal da Bahia (UFBA), Av. Reitor Miguel Calmon, S/N, Vale do Canela, Salvador, BA CEP 40140-100 Brazil; 2 Laboratório de Biotecnologia e Genética (LABIOGENE), Instituto Multidisciplinar em Saúde – Campus Anísio Teixeira (IMS/CAT), Universidade Federal da Bahia (UFBA), Rua Rio de Contas, Quadra 17, Nº 58, Bairro Candeias, Vitória da Conquista, BA CEP 45029-094 Brazil; 3 Departamento de Ciências da Vida, Universidade do Estado da Bahia (UNEB), Rua Silveira Martins, Bairro Cabula, Salvador, BA CEP 41150-000 Brazil; 4 Laboratório de Genética Molecular e Celular (LGMC), Departamento de Biologia Geral, Instituto de Ciências Biológicas (ICB), Universidade Federal de Minas Gerais (UFMG), Avenida Antonio Carlos, 6627, Pampulha, Belo Horizonte, MG Brazil

**Keywords:** *Corynebacterium pseudotuberculosis*, Caseous lymphadenitis, Sheep, Goat, Antigens, Virulence factors, Pathogenesis, Bovine fetal serum

## Abstract

**Objective:**

Previous works defining antigens that might be used as vaccine targets against *Corynebacterium pseudotuberculosis*, which is the causative agent of sheep and goat caseous lymphadenitis, have focused on secreted proteins produced in a chemically defined culture media. Considering that such antigens might not reflect the repertoire of proteins expressed during infection conditions, this experiment aimed to investigate the membrane-associated proteins with pathogenic potential expressed by *C. pseudotuberculosis* grown directly in animal serum.

**Results:**

Its membrane-associated proteins have been extracted using an organic solvent enrichment methodology, followed by LC–MS/MS and bioinformatics analysis for protein identification and classification. The results revealed 22 membrane-associated proteins characterized as potentially pathogenic. An interaction network analysis indicated that the four potentially pathogenic proteins ciuA, fagA, OppA4 and OppCD were biologically connected within two distinct network pathways, which were both associated with the ABC Transporters KEGG pathway. These results suggest that *C. pseudotuberculosis* pathogenesis might be associated with the transport and uptake of nutrients; other seven identified potentially pathogenic membrane proteins also suggest that pathogenesis might involve events of bacterial resistance and adhesion. The proteins herein reported potentially reflect part of the protein repertoire expressed during real infection conditions and might be tested as vaccine antigens.

**Electronic supplementary material:**

The online version of this article (10.1186/s13104-018-3180-5) contains supplementary material, which is available to authorized users.

## Introduction

*Corynebacterium pseudotuberculosis* is the causative agent of caseous lymphadenitis in sheep and goats, which is an infectious disease responsible for a high level of economic losses in the livestock sector [1]. During the years between 1972 and 2011, at least 39 vaccine models against *C. pseudotuberculosis* were proposed by researchers worldwide; however, no product presented satisfactory effectiveness, offering complete protection to the animals in a herd [2].

Recently, efforts have been made to characterize the bacterial exoproteome and discover novel secreted antigens for use as vaccine candidates against *C. pseudotuberculosis* infection. In particular, the use of high-throughput proteomic approaches allowed the identification of more than 100 extracellular proteins [3–8]. Albeit such advances, those results might not reflect the repertoire of proteins expressed during infection conditions, since all works used synthetic and chemically defined culture media, and did not investigate the membrane proteins, which might involve several virulence factors important for bacterial infection. Thus, this study aimed to investigate the membrane-associated proteins with pathogenic potential expressed by *C. pseudotuberculosis* grown directly in animal serum.

## Main text

### Bacterial strain and growth conditions

All the chemicals and other reagents were purchased from Sigma-Aldrich (St. Louise, MO, USA) unless otherwise stated. Strain VD57 of *C. pseudotuberculosis* was used in this study, which has been previously used as the virulent reference strain [6, 7]. The bacteria were routinely maintained in Brain Heart Infusion (BHI) broth at 37 °C. For this proteomic study, commercial bovine fetal serum (BFS) was used as integral culture medium, heated at 56 °C for 30 min prior to use to inactivate complement system proteins [8]. An overnight BHI culture (24 h) of *C. pseudotuberculosis* was inoculated in triplicate (1:10) separately into 150 mL of pre-warmed BFS and incubated at 37 °C, with agitation at 150 rpm. Growth curves were monitored by optical density (OD) at 595 nm, and 100 mL of bacterial culture was retrieved at the mid-exponential growth phase (OD595 nm = 0.7). The bacterial pellets were recovered by centrifugation at 6000 rpm for 20 min at 4 °C, then washed three times with sterile phosphate-buffered saline (PBS), and the final pellets were stored at − 70 °C until used for membrane protein extraction.

### Membrane protein extraction

Firstly, a delipidation step was performed to remove most of the lipoarabinomannans (LAMs) and phosphatidylinositol mannosides (PIMs), as previously described [9, 10]. The final pellets were subjected to a low-hydrophobicity membrane protein extraction protocol [11], using 30 volumes of 9% 1-butanol for 3 h with shaking (150 rpm) at room temperature, followed by 6000 rpm for 20 min at 4 °C. The procedure was applied twice more, and the supernatants of each extraction were dried by rota-evaporation (50 °C). The resulting protein extracts from each replicate were resuspended in sterile water, and their protein concentrations were determined by the Lowry method (DC-reagent Kit, Bio-Rad Laboratories) using bovine serum albumin (BSA) as a standard.

### Liquid chromatography and tandem mass spectrometry (LC–MS/MS)

The three protein extracts were pooled together for homogenization, then shared into six equal replicates for mass spectrometry analysis, as previously described [12]. Prior to mass spectrometry analysis, aliquots of 150 µg protein of each replicate were subjected to overnight acetone precipitation at − 20 °C to remove any contaminants. Protein precipitates were centrifuged at 13,000 rpm for 20 min at 4 °C, and after the acetone was removed, the resulting pellets were resuspended in 100 µL of NH_4_HCO_3_ (50 mM, pH 9.7). The sample preparation and the mass spectrometry analysis were performed according to a previously described protocol [13]. Briefly, the peptide separation was performed by liquid chromatography (nanoACQUITY UPLC™, Waters Corporation, Milford, MA, USA) and the mass detection of peptides was performed on a hybrid quadrupole-time-of-flight mass spectrometer (Q-TOF micro, Micromass, Alliedscienpro, Quebec, Canada) equipped with a nanoelectrospray Z spray source. Data from were processed using MassLynx (v 4.1, Waters Corporation), and the raw files from the Q-TOF micro (MS/MS data) were converted into the PKL (peak list) format; the files of the six replicates were merged into one file for the database search, which is provided as Additional file 1.

Searches employing MS/MS data were performed using the open-source software X!Tandem (www.thegpm.org/tandem/) and the specific database for *C. pseudotuberculosis* [14]; the search parameters included the enzyme entry set for trypsin, peptide tolerance of 100 ppm, fragment mass tolerance of 1.2 Da and a maximum of one missed cleavage. Cysteine carbamido methylation and methionine oxidation were set as fixed and variable modifications, respectively. Search results were validated through the Log of the E score, and peptide spectrum matches were considered significant when the Log expectation value reported by X!Tandem was smaller than − 3, meaning that the probability of a peptide match being a false positive is less than 0.005.

### Bioinformatics analysis

The bioinformatics analysis used sequences and accession numbers from the non-redundant reference strains C231, Cp267 or E19. The identified proteins underwent prediction of subcellular localization in the CELLO2GO web server (http://cello.life.nctu.edu.tw/cello2go/), with the E-value set at 0.001 [15]. All the proteins were also subjected to prediction of protein topology in the TOPCONS web server (http://topcons.cbr.su.se/), which identifies transmembrane and signal peptide regions [16]. The proteins that were in silico classified as exclusively intracellular or that presented no transmembrane-helix or signal peptides were excluded from further predictions since they were not considered to be membrane associated. The pathogenic potential of the selected proteins was determined using the MP3 web server (http://metagenomics.iiserb.ac.in/mp3/), with a threshold value set at − 0.2 [17].

The sequences were subjected to protein–protein interaction analysis using the STRING web server (http://string-db.org/) [18]. STRING was used in multiple sequences mode, and only interactions with the highest confidence levels (> 0.900) were kept, as recommended by the authors [19]. The interaction network was constructed with the whole set of target proteins, with the maximum number of interactors to show set at 110 proteins (five interactors per target protein), and the final metabolic pathway maps involving the pathogenic target proteins were searched in the Kyoto Encyclopedia of Genes and Genomes (KEGG) database as previously described [20].

### Results

*Corynebacterium pseudotuberculosis* strain VD57 has been directly cultivated in bovine fetal serum, and its membrane-associated proteins have been extracted using an organic solvent enrichment methodology, followed by LC–MS/MS analysis for protein identification. One hundred and sixteen proteins were identified by this methodology and in silico characterized according to their subcellular localization, topology and pathogenic potential (Additional file 2). Bioinformatics analysis revealed that 35 sequences were classified as membrane, cell wall or extracellular proteins and presented at least one transmembrane-helix or a signal peptide. The in silico prediction of potentially pathogenic proteins revealed that 22 of the identified membrane-associated proteins might be associated with *C. pseudotuberculosis* pathogenesis, and 11 are as yet uncharacterized proteins. The final list of identified membrane-associated proteins with predicted pathogenic potential is presented in Table 1.

**Table 1:** Membrane-associated proteins with predicted pathogenic potential expressed by *Corynebacterium pseudotuberculosis* grown in bovine fetal serum

#	Protein names	Gene names
1	Iron ABC transporter substrate-binding protein	ciuA CpC231_0982
2	FagA protein	fagA CpC231_0028
3	Oligopeptide-binding protein oppA	oppA4 CpC231_1167
4	Oligopeptide transport ATP-binding protein OppD	oppCD2 CpC231_2060
5	Mycosubtilin synthase subunit B	mycB CpC231_1794
6	ESX-3 secretion system protein eccC3	eccC3 CpC231_0404
7	ABC transporter inner membrane protein	CpC231_1467
8	Transmembrane transport protein MmpL	mmpL CpC231_1939
9	LpqU family protein	lpqU CpC231_0712
10	Surface antigen	CpC231_0173
11	Copper resistance D domain-containing protein/Cytochrome c oxidase caa3 assembly factor (Caa3_CtaG)	copD CpC231_1627
12	Uncharacterized protein	CpC231_0196
13	Uncharacterized protein	CpC231_0252
14	Uncharacterized protein	CpC231_1761
15	Uncharacterized protein	CpC231_1862
16	Uncharacterized protein	CpC231_1904
17	Uncharacterized protein	CpC231_2052
18	Uncharacterized protein	CpC231_0195
19	Uncharacterized protein	CpE19_0622
20	Uncharacterized protein	CpC231_0905
21	Uncharacterized protein	CpE19_1910
22	UPF0182 protein CpC231_0555	CpC231_0555

**Fig. 1:**
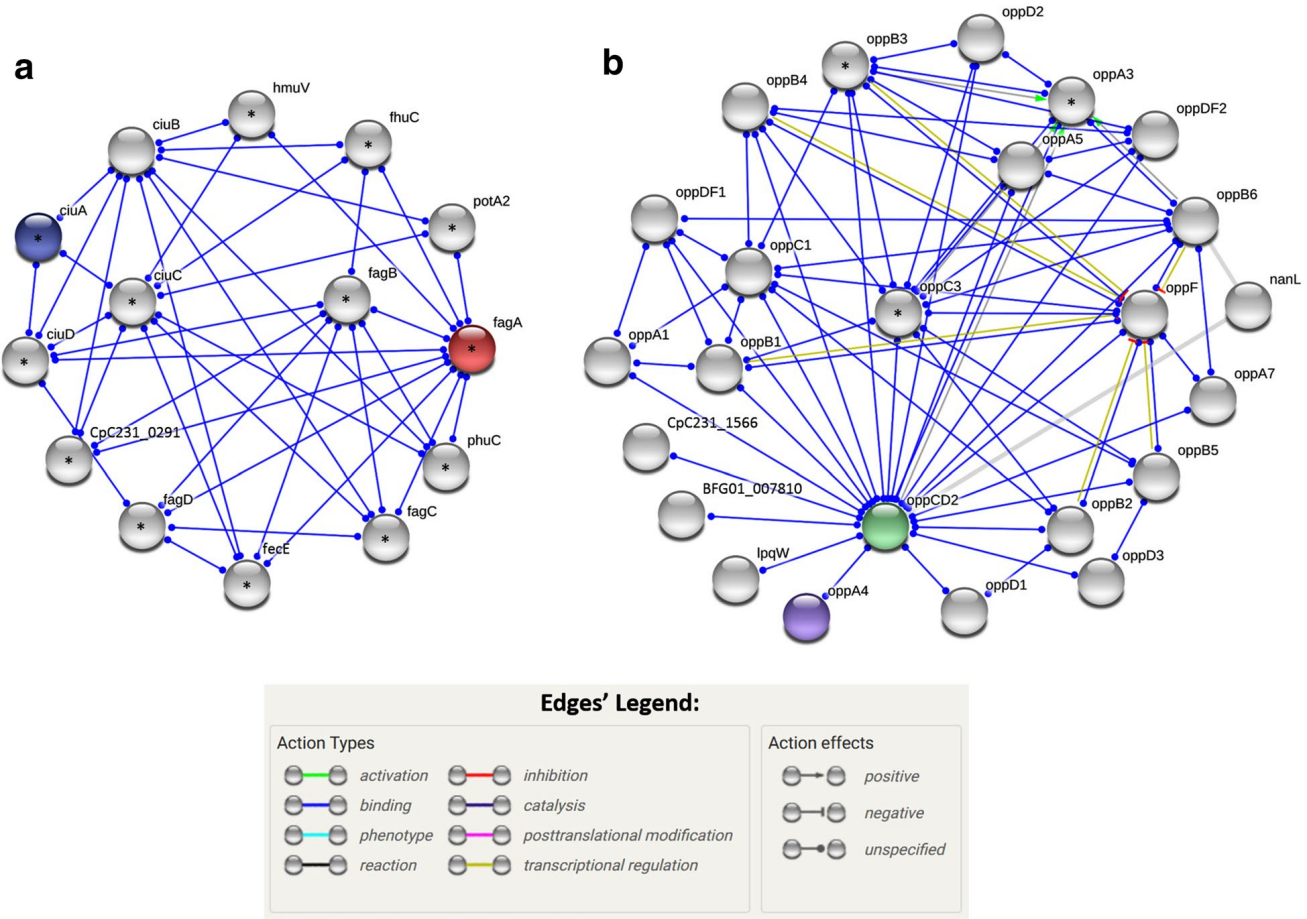
Protein–protein interaction network constructed with pathogenic membrane proteins expressed by *C. pseudotuberculosis* grown in animal serum. **a** Interaction network connecting the proteins CiuA and FagA, constructed with 12 interactor proteins and 39 interaction edges with the highest confidence levels (> 0.900). **b** Interaction network connecting the proteins oppCD2 and oppA4, constructed with 22 interactor proteins and 68 interaction edges with highest confidence levels (> 0.900). Protein nodes in grey color represent protein interactors that were automated included by String server to complete the networks. Edges represent protein–protein associations and the type of molecular action. Protein nodes with asterisks (*) are associated with the ABC Transporters KEGG pathway

The existence of cooperative work among the 22 target pathogenic proteins was investigated using the STRING server to construct interaction networks. The results indicated that the proteins CiuA and FagA are biologically connected in the same network, which was constructed with 12 interactor proteins and 39 interaction edges with the highest confidence levels (> 0.900), and was associated with the ABC Transporters KEGG pathway (Fig. 1a). The proteins oppCD2 and oppA4 were biologically connected in another interaction network constructed with 22 interactor proteins and 68 interaction edges with highest confidence levels (> 0.900); three interactor proteins were also associated with the ABC Transporters KEGG pathway (Fig. 1b).

### Discussion

The presented results indicate that the combination of organic solvent enrichment methodology with LC–MS/MS and bioinformatics analysis was effective in identifying 22 potentially pathogenic membrane associated proteins. The high number (50%) of potentially pathogenic proteins that are still uncharacterized clearly demonstrates that a greater effort is needed to investigate the virulence determinants of *C. pseudotuberculosis*. Among the 11 identified well characterized antigens, only the proteins ciuA, fagA, OppA4 and OppCD2, which were associated with the ABC Transporters KEGG pathway (Fig. 1a, b, respectively), have consistent scientific evidence of participation in *C. pseudotuberculosis* pathogenesis.

The CiuA protein is expressed by the operon ciuABCDE (ABC-type transporter and siderophore biosynthesis-related proteins) [21–23], and an experimental work using an iron-acquisition-deficient mutant strain of *C. pseudotuberculosis*, which had the ciuA gene disrupted, revealed that such strain had reduced intracellular viability during infection in murine model. These results suggested that siderophores in such intracellular bacteria improve cell viability by helping the pathogen to strip iron from chelators such as transferrin, lactoferrin and hemoglobin-haptoglobin complexes [24]. The identification of FagA in this study, as well as its connection with CiuA in the same interaction network, might indicate a possible complementation of functions between the two operons ciuABCDE and FagABCD for the uptake of iron by *C. pseudotuberculosis*. This finding is in accordance with the experimental evidence reported in a previous study using a recombinant *C. pseudotuberculosis* strain carrying a fagA-lacZ fusion, which indicated that the putative fagABC operon was highly expressed iron-limited media and that this operon was regulated by iron concentration in vitro. Another fagB(C) mutant showed reduced virulence compared to wild-type in a goat model of caseous lymphadenitis, indicating that the expression of the fag genes in the host also appears to contribute to virulence [25].

The oppA4 and oppCD2 proteins are expressed by the operon oppBCDA (oligopeptide permease) and are located in the plasma membrane, presenting functions generally related to cell nutrition and the uptake of peptides from the extracellular environment. An experiment using an OppD-deficient *C. pseudotuberculosis* strain demonstrated that this mutant presented a reduced ability to adhere to and infect macrophages compared to the wild-type bacteria [26]. Transcriptomics experiments have also demonstrated that the in vitro replication of *C. pseudotuberculosis* is decreased when it is grown under stressful conditions (acid, osmotic and thermal shock stresses), possibly due to the repression of the opp operon genes [27].

Concerning the other potentially pathogenic proteins identified in this study (Table 1), they did not have the function experimentally studied in *C. pseudotuberculosis*, but functional information from the respective heterologous proteins in other bacterial species revealed that some of them might also contribute to host–pathogen interactions and microbial virulence, as briefly discussed below.

Mycosubtilin synthase subunit B (mycB) integrates the gene cluster responsible for the biosynthesis of mycosubtilin, which has been demonstrated to present in vitro hemolytic activity [28]; the ESX-3 secretion system protein (eccC3) is a type VII secretion system that is essential for bacterial viability and is involved in iron uptake [29]. The transmembrane transport protein MmpL (mmpL) is associated with survival in macrophages and drug resistance [30], as well as with bacterial viability and virulence [31]; the lpqU family protein (lpqU) might play a role in bacterial viability and resistance [32, 33]; the copper resistance D domain-containing protein (copD) integrates a cluster of copper tolerance genes that have the main function of protecting bacteria from copper intoxication [34]. According to Uniprot annotation, the surface antigen (CpC231_0173) presents an adhesion domain, which is found in adhesin proteins, and the ABC transporter inner membrane protein (CpC231_1467) belongs to the ABC-4 integral membrane protein family and presents a permease protein domain.

In conclusion, as growing *C. pseudotuberculosis* directly in animal serum exposes the bacteria to real host constituents, the membrane proteins reported herein potentially reflect part of the protein repertoire expressed during sheep and goat caseous lymphadenitis, and might contribute for the future development of a satisfactory vaccine model. Based on the identification of potentially pathogenic antigens and protein–protein interaction network, it is suggested that *C. pseudotuberculosis* pathogenesis might be associated with the transport and uptake of nutrients, bacterial resistance, cell viability and adhesion.

### Limitations

Although this work provides a list of promising and relevant membrane-associated antigens of *C. pseudotuberculosis* grown in animal serum, that can be used in future vaccine tests, without any further functional characterization the overall nature of the present work will be only a preliminary and descriptive one. Thus, experimental validation of the in silico results are required to confirm that the identified antigens are really needed for bacterial pathogenesis and reflect part of the protein repertoire expressed under real infection conditions.

## Additional files

**Additional file 1.** Mass spectrometry data of LC–MS/MS analysis in Pkl format.

**Additional file 2.** List of identified proteins in LC–MS/MS analysis and respective bioinformatics analysis.

## Abbreviations

BHI: Brain Heart Infusion
BFS: bovine fetal serum
OD: optical density
PBS: phosphate-buffered saline
LAMs: lipoarabinomannans
PIMs: phosphatidylinositol mannosides
BSA: bovine serum albumin
LC–MS/MS: liquid chromatography and tandem mass spectrometry
KEGG: Kyoto Encyclopedia of Genes and Genomes
